# *N*,*N*′-(Biphenyl-2,2′-di­yl)bis­(furan-2-carboxamide)

**DOI:** 10.1107/S1600536809021874

**Published:** 2009-06-13

**Authors:** Chin Hui Kee, Noel F. Thomas, Azhar Ariffin, Khalijah Awang, Seik Weng Ng

**Affiliations:** a Department of Chemistry, University of Malaya, 50603 Kuala Lumpur, Malaysia

## Abstract

The title mol­ecule, C_22_H_16_N_2_O_4_, is a 2,2′-disubstituted biphenyl whose phenyl­ene rings are rotated by 66.5 (1)° so as to avoid repulsion by the substituents. Only one of the two amide –NH– fragments engages in hydrogen bonding, and this inter­acts with the amido –C(=O)– acceptor of an inversion-related mol­ecule to generate a hydrogen-bonded dimer.

## Related literature

The Heck reaction produces the desired stilbene along with a symmetrical biaryl owing to homocoupling of the aryl halide reactant. For the synthesis of stilbene carboxamides synthesized by using radical cations in a modified Heck reaction; see: Thomas *et al.* (2008 ▶).
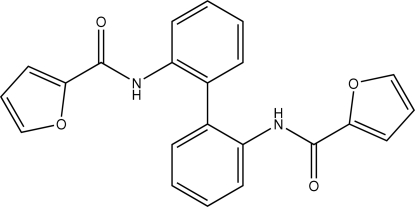


## Experimental

### 

#### Crystal data

C_22_H_16_N_2_O_4_
*M* *_r_* = 372.37
Triclinic, 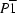

*a* = 8.1784 (2) Å
*b* = 10.1399 (2) Å
*c* = 11.1475 (2) Å
α = 99.938 (1)°
β = 107.521 (1)°
γ = 92.439 (1)°
*V* = 863.92 (3) Å^3^
*Z* = 2
Mo *K*α radiation
μ = 0.10 mm^−1^
*T* = 100 K
0.30 × 0.25 × 0.20 mm

#### Data collection

Bruker SMART APEX diffractometer
Absorption correction: none
6080 measured reflections
3836 independent reflections
3349 reflections with *I* > 2σ(*I*)
*R*_int_ = 0.015

#### Refinement

*R*[*F* ^2^ > 2σ(*F* ^2^)] = 0.037
*wR*(*F* ^2^) = 0.111
*S* = 1.03
3836 reflections
261 parameters
2 restraints
H atoms treated by a mixture of independent and constrained refinement
Δρ_max_ = 0.33 e Å^−3^
Δρ_min_ = −0.27 e Å^−3^

### 

Data collection: *APEX2* (Bruker, 2008 ▶); cell refinement: *SAINT* (Bruker, 2008 ▶); data reduction: *SAINT*; program(s) used to solve structure: *SHELXS97* (Sheldrick, 2008 ▶); program(s) used to refine structure: *SHELXL97* (Sheldrick, 2008 ▶); molecular graphics: *X-SEED* (Barbour, 2001 ▶); software used to prepare material for publication: *publCIF* (Westrip, 2009 ▶).

## Supplementary Material

Crystal structure: contains datablocks global, I. DOI: 10.1107/S1600536809021874/tk2474sup1.cif

Structure factors: contains datablocks I. DOI: 10.1107/S1600536809021874/tk2474Isup2.hkl

Additional supplementary materials:  crystallographic information; 3D view; checkCIF report

## Figures and Tables

**Table 1:** Hydrogen-bond geometry (Å, °)

*D*—H⋯*A*	*D*—H	H⋯*A*	*D*⋯*A*	*D*—H⋯*A*
N2—H2⋯O2^i^	0.88 (1)	2.12 (1)	2.970 (1)	165 (1)

Symmetry code: (i) 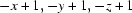.

**Fig. 1.**
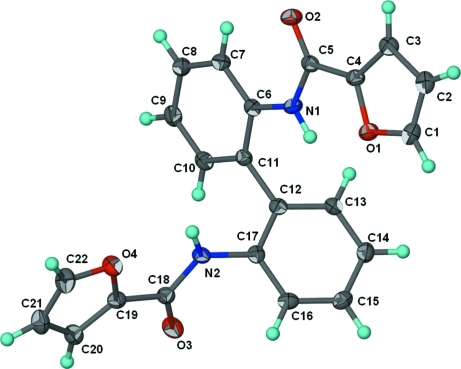
Thermal ellipsoid plot (Barbour, 2001) of C22H16N2O4 at the 70% probability level. Hydrogen atoms are drawn as spheres of arbitrary radiius.

**Fig. 2.**
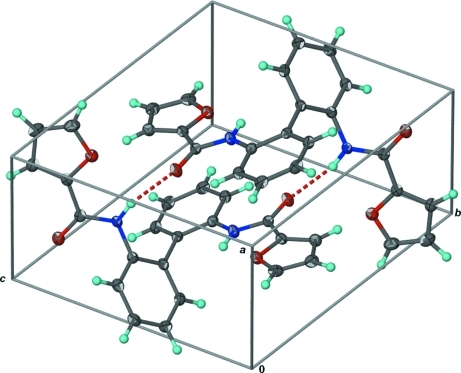
Hydrogen-bonded dimer in the crystal structure of (I). Red dashed lines represent N-H···O hydrogen bonds.

## supplementary crystallographic information

### Experimental

*N*-(2-Iodophenyl)furan-2-carboxamide (1.51 g, 4.81 mmol) was dissolved in DMF (30 ml) under a nitrogen atmosphere. The solution was heated to 393 K. Palladium acetate (0.01 g, 0.05 mmol) was added followed by triethylamine (2.4 ml, 0.2 mol) and 4-methoxystyrene (0.83 g, 6.16 mmol). The mixture was heated for 48 h. The solution was cooled and then mixed with saturated sodium chloride. The organic compound was extracted with ethylacetate. The ethylacetate solution was dried with sodium sulfate. The solvent was evaporated and the product purified by column chromatography (0.12 g, 10% yield). Single crystals were obtained by recrystallization from hexane/chloroform.

### Refinement

Carbon-bound H atoms were placed in calculated positions (C—H 0.95 Å) and were included in the refinement in the riding model approximation with *U*(H) set to 1.2*U*_eq_(C). The nitrogen-bound H atoms were located in a difference Fourier map, and were refined with a restraint of N–H 0.88±0.01 Å

### Crystal data

**Table:**

C_22_H_16_N_2_O_4_	*Z* = 2
*M**_r_* = 372.37	*F*(000) = 388
Triclinic, *P*1	*D*_x_ = 1.431 Mg m^−^^3^
Hall symbol: -P 1	Mo *K*α radiation, λ = 0.71073 Å
*a* = 8.1784 (2) Å	Cell parameters from 3508 reflections
*b* = 10.1399 (2) Å	θ = 2.6–28.3°
*c* = 11.1475 (2) Å	µ = 0.10 mm^−^^1^
α = 99.938 (1)°	*T* = 100 K
β = 107.521 (1)°	Block, colorless
γ = 92.439 (1)°	0.30 × 0.25 × 0.20 mm
*V* = 863.92 (3) Å^3^	

### Data collection

**Table:**

Bruker SMART APEX diffractometer	3349 reflections with *I* > 2σ(*I*)
Radiation source: fine-focus sealed tube	*R*_int_ = 0.015
graphite	θ_max_ = 27.5°, θ_min_ = 2.0°
ω scans	*h* = −10→10
6080 measured reflections	*k* = −12→13
3836 independent reflections	*l* = −14→14

### Refinement

**Table:**

Refinement on *F*^2^	Primary atom site location: structure-invariant direct methods
Least-squares matrix: full	Secondary atom site location: difference Fourier map
*R*[*F*^2^ > 2σ(*F*^2^)] = 0.037	Hydrogen site location: inferred from neighbouring sites
*wR*(*F*^2^) = 0.111	H atoms treated by a mixture of independent and constrained refinement
*S* = 1.03	*w* = 1/[σ^2^(*F*_o_^2^) + (0.0627*P*)^2^ + 0.3539*P*] where *P* = (*F*_o_^2^ + 2*F*_c_^2^)/3
3836 reflections	(Δ/σ)_max_ = 0.001
261 parameters	Δρ_max_ = 0.33 e Å^−^^3^
2 restraints	Δρ_min_ = −0.27 e Å^−^^3^

### Fractional atomic coordinates and isotropic or equivalent isotropic displacement parameters (Å^2^)

**Table:**

	*x*	*y*	*z*	*U*_iso_*/*U*_eq_	
O1	0.37952 (12)	0.28756 (9)	0.22024 (9)	0.0174 (2)	
O2	0.32912 (12)	0.61791 (9)	0.36569 (9)	0.0186 (2)	
O3	0.42615 (12)	0.02447 (10)	0.82668 (9)	0.0213 (2)	
O4	0.80281 (12)	0.23741 (9)	0.86550 (9)	0.0191 (2)	
N1	0.29107 (14)	0.41257 (11)	0.42005 (10)	0.0156 (2)	
H1	0.295 (2)	0.3271 (10)	0.3921 (17)	0.028 (5)*	
N2	0.47787 (14)	0.16551 (11)	0.70037 (10)	0.0148 (2)	
H2	0.5491 (17)	0.2298 (13)	0.6951 (15)	0.016 (4)*	
C1	0.40891 (17)	0.24580 (13)	0.10565 (13)	0.0190 (3)	
H1a	0.4304	0.1570	0.0749	0.023*	
C2	0.40293 (17)	0.34894 (14)	0.04255 (13)	0.0190 (3)	
H2a	0.4180	0.3456	−0.0391	0.023*	
C3	0.36958 (16)	0.46370 (13)	0.12235 (12)	0.0173 (3)	
H3	0.3581	0.5517	0.1044	0.021*	
C4	0.35755 (16)	0.42216 (12)	0.22885 (12)	0.0155 (3)	
C5	0.32496 (16)	0.49452 (12)	0.34438 (12)	0.0145 (2)	
C6	0.24862 (16)	0.44495 (13)	0.53444 (12)	0.0144 (2)	
C7	0.23946 (17)	0.57726 (13)	0.59222 (12)	0.0169 (3)	
H7	0.2603	0.6501	0.5537	0.020*	
C8	0.19978 (17)	0.60133 (13)	0.70591 (12)	0.0182 (3)	
H8	0.1950	0.6913	0.7455	0.022*	
C9	0.16695 (17)	0.49624 (14)	0.76291 (12)	0.0192 (3)	
H9	0.1386	0.5138	0.8404	0.023*	
C10	0.17609 (17)	0.36502 (13)	0.70536 (12)	0.0174 (3)	
H10	0.1539	0.2928	0.7443	0.021*	
C11	0.21726 (16)	0.33732 (12)	0.59167 (12)	0.0142 (2)	
C12	0.21741 (16)	0.19410 (12)	0.53056 (12)	0.0142 (3)	
C13	0.08880 (17)	0.13778 (13)	0.41514 (12)	0.0164 (3)	
H13	0.0094	0.1937	0.3731	0.020*	
C14	0.07515 (17)	0.00244 (13)	0.36117 (12)	0.0182 (3)	
H14	−0.0121	−0.0337	0.2825	0.022*	
C15	0.18976 (17)	−0.08003 (13)	0.42285 (12)	0.0178 (3)	
H15	0.1790	−0.1734	0.3877	0.021*	
C16	0.31983 (16)	−0.02613 (13)	0.53571 (12)	0.0161 (3)	
H16	0.3991	−0.0826	0.5770	0.019*	
C17	0.33501 (16)	0.11065 (12)	0.58905 (12)	0.0141 (2)	
C18	0.52125 (16)	0.10827 (12)	0.80562 (12)	0.0150 (3)	
C19	0.69553 (16)	0.15138 (12)	0.89706 (12)	0.0155 (3)	
C20	0.77797 (18)	0.11564 (13)	1.01001 (13)	0.0195 (3)	
H20	0.7319	0.0577	1.0529	0.023*	
C21	0.94791 (18)	0.18268 (15)	1.05134 (13)	0.0226 (3)	
H21	1.0380	0.1779	1.1272	0.027*	
C22	0.95558 (18)	0.25413 (15)	0.96175 (13)	0.0226 (3)	
H22	1.0546	0.3090	0.9652	0.027*	

### Atomic displacement parameters (Å^2^)

**Table:**

	*U*^11^	*U*^22^	*U*^33^	*U*^12^	*U*^13^	*U*^23^
O1	0.0214 (5)	0.0138 (4)	0.0181 (4)	0.0017 (4)	0.0076 (4)	0.0032 (3)
O2	0.0226 (5)	0.0136 (4)	0.0217 (5)	0.0018 (4)	0.0085 (4)	0.0058 (4)
O3	0.0197 (5)	0.0241 (5)	0.0206 (5)	−0.0031 (4)	0.0051 (4)	0.0095 (4)
O4	0.0162 (5)	0.0212 (5)	0.0196 (5)	−0.0015 (4)	0.0041 (4)	0.0065 (4)
N1	0.0211 (6)	0.0113 (5)	0.0162 (5)	0.0023 (4)	0.0082 (4)	0.0031 (4)
N2	0.0136 (5)	0.0140 (5)	0.0169 (5)	−0.0005 (4)	0.0037 (4)	0.0057 (4)
C1	0.0176 (6)	0.0193 (6)	0.0187 (6)	−0.0010 (5)	0.0066 (5)	−0.0011 (5)
C2	0.0166 (6)	0.0231 (6)	0.0170 (6)	−0.0003 (5)	0.0058 (5)	0.0029 (5)
C3	0.0160 (6)	0.0178 (6)	0.0185 (6)	0.0005 (5)	0.0056 (5)	0.0050 (5)
C4	0.0143 (6)	0.0132 (6)	0.0185 (6)	0.0005 (5)	0.0036 (5)	0.0044 (5)
C5	0.0124 (5)	0.0151 (6)	0.0158 (6)	0.0013 (4)	0.0031 (5)	0.0050 (5)
C6	0.0132 (6)	0.0158 (6)	0.0140 (6)	0.0022 (4)	0.0031 (5)	0.0039 (5)
C7	0.0176 (6)	0.0147 (6)	0.0177 (6)	0.0030 (5)	0.0038 (5)	0.0044 (5)
C8	0.0182 (6)	0.0163 (6)	0.0176 (6)	0.0056 (5)	0.0026 (5)	0.0012 (5)
C9	0.0198 (6)	0.0230 (7)	0.0143 (6)	0.0049 (5)	0.0048 (5)	0.0031 (5)
C10	0.0178 (6)	0.0192 (6)	0.0157 (6)	0.0015 (5)	0.0044 (5)	0.0060 (5)
C11	0.0120 (6)	0.0143 (6)	0.0148 (6)	0.0019 (4)	0.0016 (5)	0.0037 (4)
C12	0.0153 (6)	0.0140 (6)	0.0150 (6)	−0.0001 (5)	0.0063 (5)	0.0047 (5)
C13	0.0178 (6)	0.0162 (6)	0.0154 (6)	0.0026 (5)	0.0044 (5)	0.0053 (5)
C14	0.0194 (6)	0.0181 (6)	0.0153 (6)	−0.0015 (5)	0.0040 (5)	0.0018 (5)
C15	0.0211 (6)	0.0139 (6)	0.0193 (6)	−0.0002 (5)	0.0089 (5)	0.0018 (5)
C16	0.0157 (6)	0.0146 (6)	0.0202 (6)	0.0034 (5)	0.0078 (5)	0.0053 (5)
C17	0.0141 (6)	0.0156 (6)	0.0137 (6)	0.0000 (5)	0.0057 (5)	0.0040 (4)
C18	0.0154 (6)	0.0147 (6)	0.0156 (6)	0.0032 (5)	0.0055 (5)	0.0032 (5)
C19	0.0163 (6)	0.0147 (6)	0.0166 (6)	0.0018 (5)	0.0064 (5)	0.0036 (5)
C20	0.0205 (7)	0.0206 (6)	0.0171 (6)	0.0020 (5)	0.0049 (5)	0.0049 (5)
C21	0.0183 (7)	0.0283 (7)	0.0178 (6)	0.0018 (5)	0.0017 (5)	0.0023 (5)
C22	0.0152 (6)	0.0277 (7)	0.0219 (7)	−0.0026 (5)	0.0032 (5)	0.0024 (5)

### Geometric parameters (Å, °)

**Table:**

O1—C1	1.3695 (16)	C8—H8	0.9500
O1—C4	1.3749 (15)	C9—C10	1.3893 (18)
O2—C5	1.2295 (15)	C9—H9	0.9500
O3—C18	1.2267 (16)	C10—C11	1.3931 (18)
O4—C22	1.3613 (16)	C10—H10	0.9500
O4—C19	1.3716 (15)	C11—C12	1.4941 (17)
N1—C5	1.3572 (16)	C12—C17	1.3957 (18)
N1—C6	1.4093 (16)	C12—C13	1.4030 (17)
N1—H1	0.875 (9)	C13—C14	1.3853 (17)
N2—C18	1.3569 (16)	C13—H13	0.9500
N2—C17	1.4311 (16)	C14—C15	1.3884 (19)
N2—H2	0.877 (9)	C14—H14	0.9500
C1—C2	1.3521 (19)	C15—C16	1.3858 (18)
C1—H1a	0.9500	C15—H15	0.9500
C2—C3	1.4267 (19)	C16—C17	1.3961 (17)
C2—H2a	0.9500	C16—H16	0.9500
C3—C4	1.3550 (18)	C18—C19	1.4765 (18)
C3—H3	0.9500	C19—C20	1.3559 (18)
C4—C5	1.4715 (18)	C20—C21	1.4255 (19)
C6—C7	1.3999 (17)	C20—H20	0.9500
C6—C11	1.4067 (17)	C21—C22	1.345 (2)
C7—C8	1.3851 (18)	C21—H21	0.9500
C7—H7	0.9500	C22—H22	0.9500
C8—C9	1.3869 (19)		
			
C1—O1—C4	106.32 (10)	C11—C10—H10	119.4
C22—O4—C19	105.90 (10)	C10—C11—C6	118.87 (12)
C5—N1—C6	129.64 (11)	C10—C11—C12	119.07 (11)
C5—N1—H1	114.1 (12)	C6—C11—C12	121.97 (11)
C6—N1—H1	116.3 (12)	C17—C12—C13	118.24 (11)
C18—N2—C17	122.19 (10)	C17—C12—C11	122.04 (11)
C18—N2—H2	118.8 (10)	C13—C12—C11	119.58 (11)
C17—N2—H2	118.2 (10)	C14—C13—C12	121.42 (12)
C2—C1—O1	110.34 (11)	C14—C13—H13	119.3
C2—C1—H1a	124.8	C12—C13—H13	119.3
O1—C1—H1a	124.8	C13—C14—C15	119.56 (12)
C1—C2—C3	106.73 (12)	C13—C14—H14	120.2
C1—C2—H2a	126.6	C15—C14—H14	120.2
C3—C2—H2a	126.6	C16—C15—C14	120.02 (12)
C4—C3—C2	106.31 (12)	C16—C15—H15	120.0
C4—C3—H3	126.8	C14—C15—H15	120.0
C2—C3—H3	126.8	C15—C16—C17	120.36 (12)
C3—C4—O1	110.30 (11)	C15—C16—H16	119.8
C3—C4—C5	131.50 (12)	C17—C16—H16	119.8
O1—C4—C5	118.19 (11)	C12—C17—C16	120.35 (11)
O2—C5—N1	125.53 (12)	C12—C17—N2	120.43 (11)
O2—C5—C4	120.77 (11)	C16—C17—N2	119.14 (11)
N1—C5—C4	113.70 (11)	O3—C18—N2	124.03 (12)
C7—C6—C11	120.02 (11)	O3—C18—C19	119.98 (12)
C7—C6—N1	122.95 (11)	N2—C18—C19	115.98 (11)
C11—C6—N1	117.03 (11)	C20—C19—O4	110.52 (11)
C8—C7—C6	119.61 (12)	C20—C19—C18	131.14 (12)
C8—C7—H7	120.2	O4—C19—C18	118.26 (11)
C6—C7—H7	120.2	C19—C20—C21	106.02 (12)
C9—C8—C7	121.07 (12)	C19—C20—H20	127.0
C9—C8—H8	119.5	C21—C20—H20	127.0
C7—C8—H8	119.5	C22—C21—C20	106.47 (12)
C8—C9—C10	119.20 (12)	C22—C21—H21	126.8
C8—C9—H9	120.4	C20—C21—H21	126.8
C10—C9—H9	120.4	C21—C22—O4	111.09 (12)
C9—C10—C11	121.23 (12)	C21—C22—H22	124.5
C9—C10—H10	119.4	O4—C22—H22	124.5
			
C4—O1—C1—C2	−1.04 (14)	C10—C11—C12—C13	109.90 (14)
O1—C1—C2—C3	0.66 (15)	C6—C11—C12—C13	−66.72 (16)
C1—C2—C3—C4	0.00 (14)	C17—C12—C13—C14	1.43 (19)
C2—C3—C4—O1	−0.65 (14)	C11—C12—C13—C14	−174.25 (11)
C2—C3—C4—C5	179.98 (13)	C12—C13—C14—C15	0.66 (19)
C1—O1—C4—C3	1.04 (14)	C13—C14—C15—C16	−1.8 (2)
C1—O1—C4—C5	−179.49 (11)	C14—C15—C16—C17	0.93 (19)
C6—N1—C5—O2	2.2 (2)	C13—C12—C17—C16	−2.34 (18)
C6—N1—C5—C4	−177.63 (12)	C11—C12—C17—C16	173.22 (11)
C3—C4—C5—O2	−11.2 (2)	C13—C12—C17—N2	174.26 (11)
O1—C4—C5—O2	169.42 (11)	C11—C12—C17—N2	−10.18 (18)
C3—C4—C5—N1	168.56 (13)	C15—C16—C17—C12	1.20 (18)
O1—C4—C5—N1	−10.77 (16)	C15—C16—C17—N2	−175.44 (11)
C5—N1—C6—C7	−1.9 (2)	C18—N2—C17—C12	133.43 (13)
C5—N1—C6—C11	178.99 (12)	C18—N2—C17—C16	−49.93 (17)
C11—C6—C7—C8	0.14 (19)	C17—N2—C18—O3	−15.7 (2)
N1—C6—C7—C8	−178.89 (12)	C17—N2—C18—C19	163.63 (11)
C6—C7—C8—C9	−0.7 (2)	C22—O4—C19—C20	0.32 (15)
C7—C8—C9—C10	0.7 (2)	C22—O4—C19—C18	−176.70 (11)
C8—C9—C10—C11	−0.1 (2)	O3—C18—C19—C20	−1.2 (2)
C9—C10—C11—C6	−0.48 (19)	N2—C18—C19—C20	179.49 (13)
C9—C10—C11—C12	−177.20 (12)	O3—C18—C19—O4	175.11 (11)
C7—C6—C11—C10	0.46 (18)	N2—C18—C19—O4	−4.22 (17)
N1—C6—C11—C10	179.55 (11)	O4—C19—C20—C21	−0.45 (15)
C7—C6—C11—C12	177.08 (11)	C18—C19—C20—C21	176.06 (13)
N1—C6—C11—C12	−3.83 (18)	C19—C20—C21—C22	0.40 (16)
C10—C11—C12—C17	−65.60 (16)	C20—C21—C22—O4	−0.22 (16)
C6—C11—C12—C17	117.78 (14)	C19—O4—C22—C21	−0.05 (15)

### Hydrogen-bond geometry (Å, °)

**Table:**

*D*—H···*A*	*D*—H	H···*A*	*D*···*A*	*D*—H···*A*
N2—H2···O2^i^	0.88 (1)	2.12 (1)	2.970 (1)	165 (1)

Symmetry codes: (i) −*x*+1, −*y*+1, −*z*+1.
